# Antibacterial Activity of Polymer Nanocomposites Incorporating Graphene and Its Derivatives: A State of Art

**DOI:** 10.3390/polym13132105

**Published:** 2021-06-26

**Authors:** Ana M. Díez-Pascual, José A. Luceño-Sánchez

**Affiliations:** Universidad de Alcalá, Facultad de Ciencias, Departamento de Química Analítica, Química Física e Ingeniería Química, Ctra. Madrid-Barcelona, Km. 33.6, 28805 Alcalá de Henares, Madrid, Spain; jose.luceno@uah.es

**Keywords:** antimicrobial activity, polymer nanocomposites, graphene, graphene oxide, reactive oxygen species, synergic effects

## Abstract

The incorporation of carbon-based nanostructures into polymer matrices is a relevant strategy for producing novel antimicrobial materials. By using nanofillers of different shapes and sizes, and polymers with different characteristics, novel antimicrobial nanocomposites with synergistic properties can be obtained. This article describes the state of art in the field of antimicrobial polymeric nanocomposites reinforced with graphene and its derivatives such as graphene oxide and reduced graphene oxide. Taking into account the vast number of articles published, only some representative examples are provided. A classification of the different nanocomposites is carried out, dividing them into acrylic and methacrylic matrices, biodegradable synthetic polymers and natural polymers. The mechanisms of antimicrobial activity of graphene and its derivatives are also reviewed. Finally, some applications of these antimicrobial nanocomposites are discussed. We aim to enhance understanding in the field and promote further work on the development of polymer-based antimicrobial nanocomposites incorporating graphene-based nanomaterials.

## 1. Introduction

Severe infections produced by pathogenic bacteria have been studied extensively for over a century. Though the evolution of novel antibiotic and other methods to hold infections has aided humans in preventing and treating many diseases over the last decades [1], recently, treatment of microbial infection has become more complex since bacteria have the ability to conform themselves against antibiotics. Thus, they can rapidly and easily mutate their genes, making their elimination difficult. For instance, *Pseudomonas aeruginosa* (*P. aeruginosa*) bacterium is one of the most frequent causes of healthcare related infections and is progressively becoming more resistant to numerous antibiotics. *Staphylococcus aureus* (*S. aureus*) is another bacterium that commonly colonizes human skin and mucosa, and can cause serious illnesses if the bacteria come into the body, such as in wound infections, endocarditis, pneumonia, blood stream infection and so forth [2]. *Escherichia coli (E. coli)* is a bacterium commonly found in the gut of warm-blooded organisms. Most strains of *E. coli* are not harmful but are part of the healthful bacterial flora in the human gut. However, some types can cause illness in humans, including diarrhea, abdominal pain, fever, and sometimes vomiting. Other types of E. coli infection can lead to urinary tract infections, respiratory illness, pneumonia, and meningitis.

Contamination by microorganisms is a critical parameter in diverse areas, such as medical devices, dental equipment, healthcare products and hygienic applications, hospital surfaces/furniture, dental restoration, water purification systems, food packaging and storage, and so forth. To solve this issue, several types of thermoplastic materials are sterilized and antisepsis by different methods, including dry or wet heating and ionic radiation, are employed. However, microorganisms can pollute these polymers when exposed to the environment. Therefore, there is an urgent need for novel polymeric material with antibacterial activity [3]. Thermoplastics are strong and able to resist repeated sterilization, high severe temperatures and chemical environments. Consequently, antimicrobial polymer composites can be employed as a strategy to circumvent hospital-acquired infections, and can be prepared either by inserting a biocide agent into the polymer bulk, i.e., during their processing or by applying surface coatings [4,5]. Furthermore, three main groups of polymer nanocomposites have been reported depending on how the nanoparticles and polymers are combined [6]: (1) non-polymer coated nanoparticles distributed in a polymer matrix, (2) nanoparticles attached to polymeric micro/nanostructures, (3) polymer-grafted nanoparticles, which form a core shell structure, dispersed inside a polymer matrix. Hybrid structures with carbon cores and polymer brushes can be formed via controlled/living polymerization as a result of “grafting to” or “grafting from” methods [7,8].

**Scheme 1:**
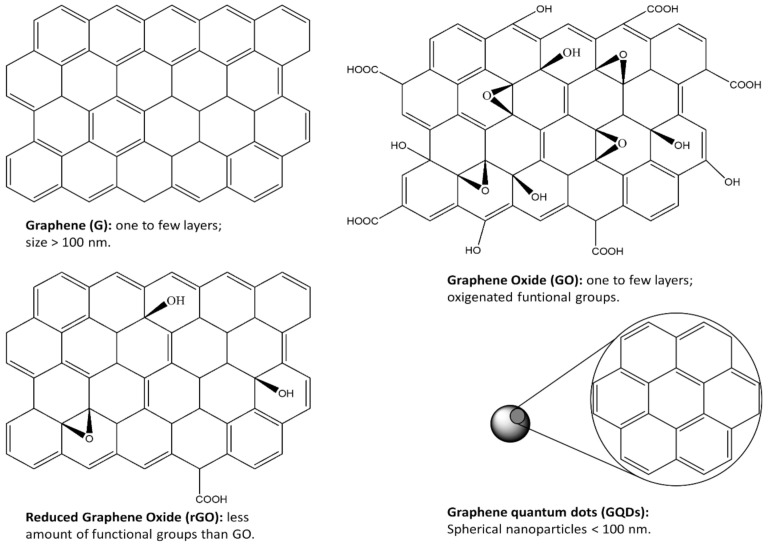
Chemical structure of graphene and its derivatives, graphene oxide (GO), reduced graphene oxide (rGO) and graphene quantum dots (GQDs).

Up until now, a wide number of antimicrobial agents, including metal nanoparticles, metal-oxide nanoparticles [9] and carbon nanomaterials [3], have been used as fillers to reinforce polymeric matrices. Recently, graphene (G) and its derivatives (Scheme 1), graphene oxide (GO), reduced graphene oxide (rGO) and graphene quantum dots (GQDs) have arisen as effective antimicrobial agents on pathogens, fungi, and bacteria [10].

Graphene is an atomically thin, 2D layer of sp^2^ carbon atoms in a honeycomb structure. It possesses a unique combination of properties including high stiffness and strength, elevated thermal and electrical conductivity, very high electron mobility, molecular barrier capability, low toxicity and so forth [11]. For these reasons, a lot of effort has been devoted to integrating G into polymeric matrices to design polymer-based nanocomposites with outstanding properties due to synergistic effects. Nonetheless, the use of pristine G has been limited due to its insolubility in aqueous media, and strong agglomeration tendency, owing to van der Waals interactions among sheets. To solve this issue, several graphene derivatives, including GO, rGO and GQDs have been used as nanofillers in polymer nanocomposites.

GO is an oxidized form of G that contains epoxides, hydroxyls and carbonyls on the basal planes and carboxylic acids on the edges (Scheme 1). Owing to the attached functional groups, it offers surface-functionalization capability, aqueous processability, amphiphilicity, biocompatibility, and the capacity to interact with tissues and biological cells [12]. GO can be partly reduced to graphene-like sheets by eliminating the oxygen-containing groups with the recovery of a conjugated structure, leading to reduced GO (rGO) sheets [13]. Recently, graphene quantum dots (GQDs) have emerged (Scheme 1), which are basically graphene spherical nanoparticles with a size of less than 100 nm. Due to their excellent properties such as high solubility in various solvents, high specific surface area, plenty of edge sites for functionalization, versatility, stable photoluminescence, chemical stability, low toxicity, and strong quantum confinement effect, GQDs are considered as a novel material for biological, opto-electronics, energy and environmental applications.

Over the last years, a vast number of reviews dealing with polymeric materials with antimicrobial activity have been published [14,15,16]. Furthermore, a few articles have reviewed the antimicrobial properties of G-based materials [17,18,19]. In this article, the antimicrobial properties of polymeric composites comprising G or its derivatives are reviewed. Due to the large number of articles in the field, only some representative examples are described. The idea is to provide the reader with a panoramic of the current state of art in this exciting and lively field, and to promote further work on the development of polymer-based antimicrobial nanocomposites. Additionally, some applications of these antimicrobial nanocomposites are discussed.

## 2. Mechanisms of Antimicrobial Activity of Graphene and Its Derivatives

**Scheme 2:**
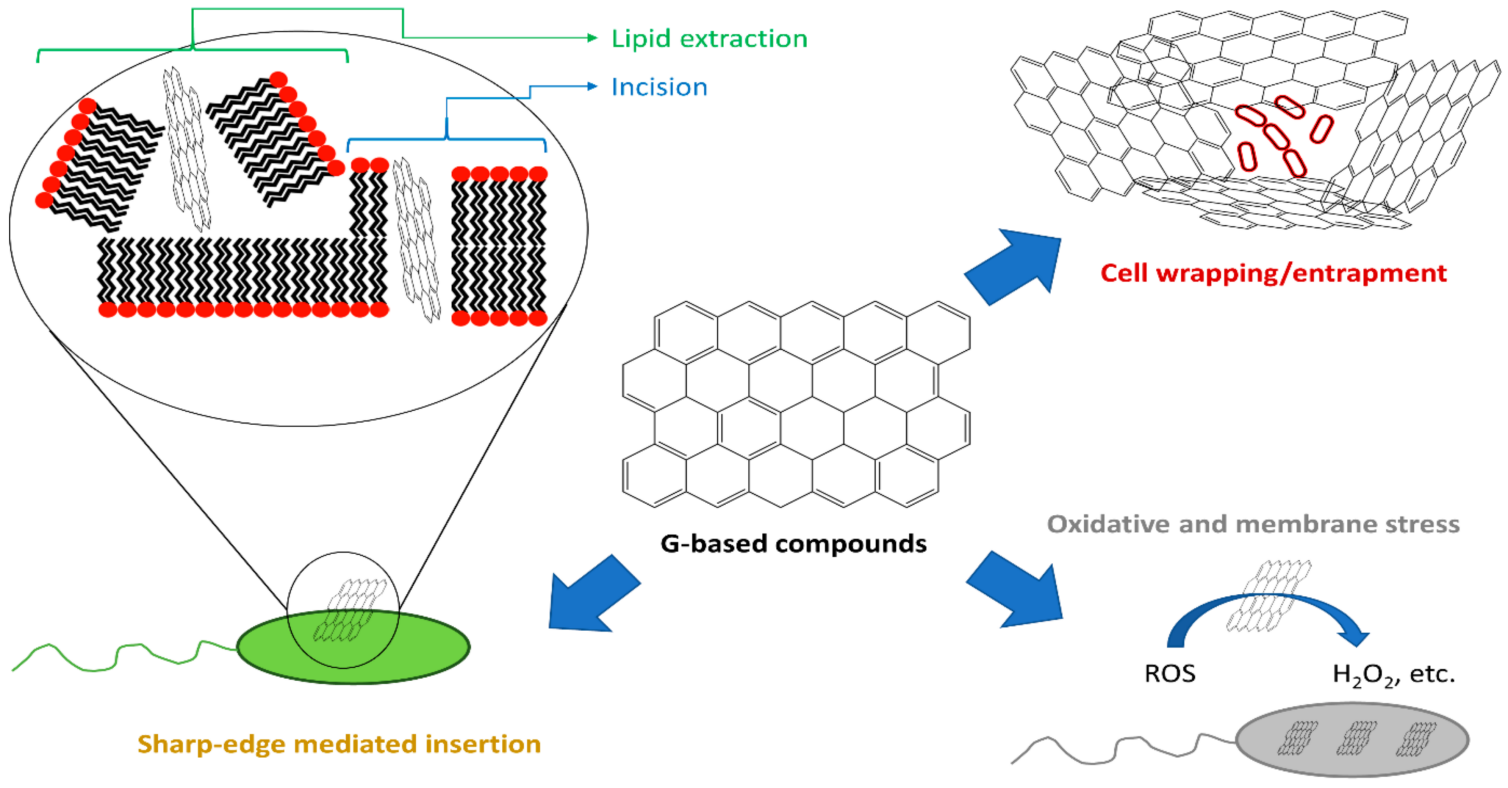
Representation of the mechanisms of antibacterial activity of G and its derivatives.

Hu et al. [20] in 2010 reported for the first time that GO possessed excellent antimicrobial activity. Since then, numerous studies have reported the antimicrobial activity of G and its derivatives [21,22,23,24]. Their antibacterial action can be ascribed to several mechanisms, including chemical and physical processes, as depicted in Scheme 2.

### 2.1. Chemical Mechanisms of Antibacterial Action

**Scheme 3:**
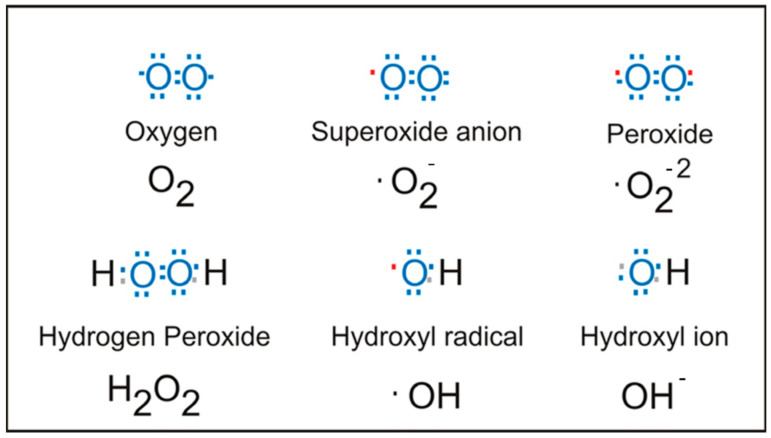
Structures of reactive oxygen species (ROS).

Among the chemical mechanism of bactericide activity is oxidative stress, which is defined as excess production of reactive oxygen species (ROS) relative to antioxidant defense. ROS are oxygen-based molecules and free radicals with high reactivity. Examples of ROS include peroxides (H_2_O_2_), superoxide (^•^O^−^_2_), hydroxyl radical (^•^OH), hydroxyl ion (OH^−^), and singlet oxygen (^1^O_2_) (Scheme 3). ROS can be harmful to cells due to oxidative damage to lipids, proteins, and DNA. Moreover, ROS are extremely destructive to organisms at high concentrations causing peroxidation of lipids, oxidation of proteins, and damage to nucleic acids, enzyme inhibition, activation of programmed cell death (PCD) pathway, and ultimately leading to the death of the cells [25].

Liu et al. [21] found that antimicrobial actions of G-based nanomaterials are derived from both membrane and oxidation stress. They proposed a three-step antimicrobial mechanism including initial cell deposition on the nanomaterial, membrane stress produced by direct contact with sharp nanosheets, and finally ROS generation that provokes oxidative stress via electron transfer route. They compared the antibacterial activity of G, GO, rGO and graphite oxide toward a bacterial model, *E. coli*. Under similar conditions, GO dispersion showed the highest antibacterial activity, followed by rGO, G, and graphite oxide. In fact, the shape, size, surface functional groups, and electronic structure of G-based nanomaterials have a strong influence on the antibacterial activity. Scanning electron microscopy (SEM) and dynamic light scattering (DLS) analyses demonstrated that GO nanostructures had the smallest average size among the four types of materials. The reduction in size causes greater defect density, hence stronger oxidative stress. Furthermore, the number of oxygen-containing functional groups conditions the antibacterial action [26]. In this regard, Zhang et al. [27] investigated the oxidation degree-dependent toxicity of GO and found that GOs with lower oxidation degrees generated higher levels of ROS and oxidative impairment by promoting the H_2_O_2_ decomposition into the hydroxyl radicals and superior direct oxidative abilities on the bacteria cells. Furthermore, theoretical calculations demonstrated that the size of the aromatic domains in the G sheets and the amount of COOH groups had a noteworthy effect on the energy barrier of the H_2_O_2_ decomposition reaction.

Nonetheless, the antibacterial activity of G derivatives does not arise only from ROS-mediated damage, but through electron transfer interaction from microbial membrane to graphene [28]. Thus, the physical contact of microorganisms with G will result in Schottky barrier formation and Fermi level alignment based on the band theory, which enables the facile transfer of electrons from bacterial membranes to graphene materials that are good electron acceptors. In this regard, functionalities also influence the electron transfer processes, particularly because GO shows more insulating character compared to pristine G (metallic) and rGO (semiconductor).

### 2.2. Physical Mechanisms of Antibacterial Action

Several physical mechanisms, including direct contact of the G sharp edges with the bacterial membrane, wrapping/entrapment of the bacterial cell and lipid extraction, have been proposed (Scheme 2). The former mechanism is also known as “insertion mode of action” or “sharp edge mediated insertion”, in which G nanosheets use their sharp edges acting as a blade to cut the bacterial cell through the cell membrane and cause cell death due to leakage of intracellular material. Akhavan and Ghaderi [23] reported that the biocidal activity of rGO is higher than that of GO for different bacterial strains. In particular, the Gram-negative *E. coli* bacteria with an outer membrane were more resistant to the cell membrane damage caused by the rGO than the Gram-positive *S. aureus* lacking the outer membrane. Moreover, rGO was more toxic to the bacteria than GO due to its more sharpened edges during the contact interaction. The nanomaterials cause cell death due to the outflow of the intracellular materials by mechanically disrupting the bacterial cell membrane. Related to the insertion mode of action is another mechanism, which postulates that the destructive effect of G on the bacterial membrane is induced by “direct contact” with the basal plane of the nanomaterial. Thus, the antibacterial effect does not depend on the sharp edges, but on the contact that occurs between the nanomaterial basal plane and the bacteria cells. Thus, screening the GO basal plane decreased its antimicrobial efficiency by lessening the degree of direct contact with the bacteria.

Another potential mechanism is the lipid extraction. Thus, Li et al. [29] reported that the G sheets could enter the bacterial cell via lipid bilayer by piercing the cell wall of bacteria with its sharp edges. Yi et al. [30] showed that the size influences the incorporation of G into the lipid bilayer. Near-perpendicular configuration to the cell wall was preferentially adopted by larger size G, while smaller nanosheets adopted a parallel conformation. The interaction between the hydrocarbon tail of the phospholipids and the flat lipophilic surface of G promotes the insertion of the G nanosheets into the cell membrane. However, Dallavalle et al. [31] reported that the smaller sheets orientate perpendicular to the lipid membrane and diffuse into the lipid, while larger ones penetrate the more lipophilic-portion of cell membrane by arranging themselves across the membrane. It was also suggested that the bactericidal activity of GO nanosheets could be a thermodynamically driven process with no need for energy consumption. Moreover, the cytotoxicity depends on the flake size. Smaller flakes are more cytotoxic and display greater cellular-internalization, hence they exert more influence on the cell functionality. In addition, the number of layers of G or its derivatives affect the antimicrobial activity. Thus, it has been proven via simulations that few-layer graphene has lower bactericide action that monolayer graphene [32]. In addition, the increase in the number of layers typically causes aggregation of graphene, which is reflected in lower interaction between the nanomaterial and the bacteria.

Another possible mechanism that causes antibacterial activity was proposed by Luan et al. [33], who reported that the lipophilic flat surface of G disrupted the protein-protein bonding in the cell membrane and led to functional failure.

Wrapping or entrapping of the bacterial cell occurs when bacteria are enclosed in G-based sheets and thus separated from their growth medium [34]. This mechanism has been observed with both G and GO nanosheets inhibiting nutrients to pass through the cell membrane, and leading to growth inhibition. G or GO nanosheets with higher lateral size hinder bacterial growth more efficiently by cell wrapping, nevertheless only in a temporary and reversible manner. Other G characteristics that influence the antibacterial activity are the specific surface area and roughness. In this regard, the larger the specific surface area, the higher the number of sites available for bacterial microbial interaction and adhesion. The rougher the surface, the larger the number of irregularities, hence more contact points with the bacteria [35], which is reflected in more efficient biocide action. Additionally, the level of dispersibility of G or GO in the medium strongly affects the antimicrobial effect [11].

## 3. Antimicrobial Polymeric Nanocomposites Incorporating Graphene-Based Nanomaterials

Polymeric nanocomposites are materials with polymers as a matrix and nanofillers as reinforcing material. Polymeric nanocomposites incorporating graphene can be prepared via different techniques such as melt compounding, solution blending, in situ polymerization, latex mixing, and electropolymerization [36]. Furthermore, different methods have been used to modify graphene and other carbon nanomaterials with polymers via covalent and non-covalent approaches such as hydrophobic interactions, π–π stacking, and van der Waals forces [37]. Taking into account the polymer matrix, different polymer/graphene nanocomposites with antibacterial activity have been developed, as will be described in the following sections.

### 3.1. Nanocomposites Based on Acrylic and Methacrylic Polymers

Many polymers derived from acrylic or methacrylic acid present antimicrobial properties. In this regard, a number of studies have investigated the effect of G addition to MAA derived polymers. For instance, Bacali et al. [38] evaluated the effect of adding GO-Ag nanoparticles to a polymethyl methacrylate (PMMA) auto-polymerizing resin. When Ag nanoparticles interact with bacteria, they agglomerate; hence their effective specific surface area decreases, leading to reduced antibacterial action [39]. However, the hybrids with 1 and 2 wt% GO-Ag showed very good antimicrobial activity against *S. aureus, E. coli* and *S. mutans.* The GO-Ag nanocomposite sheets likely wrap around the microorganism in direct contact, aided by hydrogen bonding with the cell membrane proteins, blocking them and causing apoptosis. Thus, there is likely a synergistic effect of both nanofillers on improving antibacterial activity, as reported for several graphene-based multicomponent composites [39].

Fiber meshes of PMMA containing GO have also been developed via pressurized gyration, and their antibacterial potential against *E. coli* was investigated [40]. Average bacterial reduction ranged from 46 to 85%, and the optimal bactericide action was found for the nanocomposite containing 8 wt% of GO. Bacterial toxicity of the nanocomposites was analyzed in terms of ROS formation.

**Figure 1:**
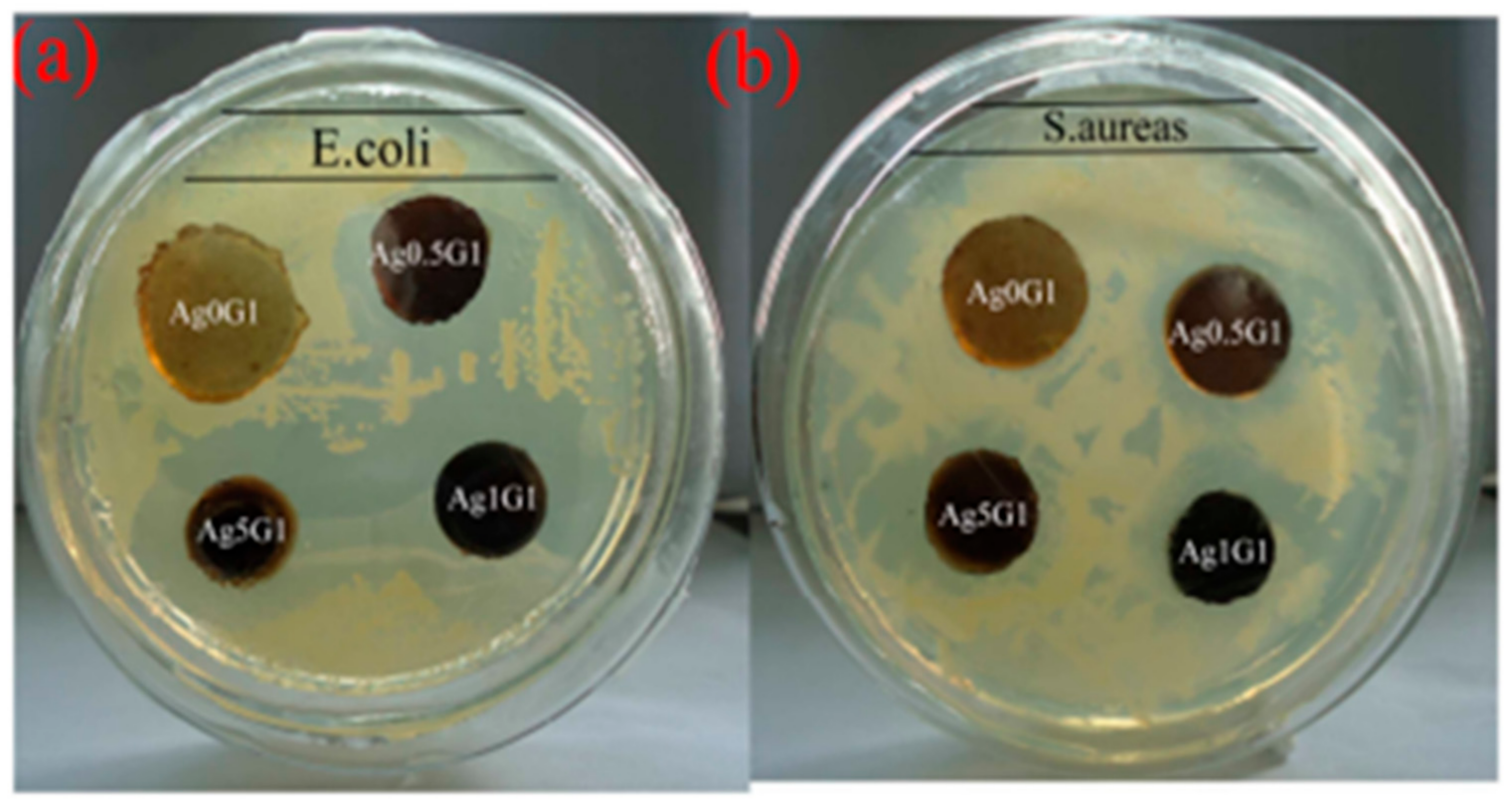
Photographs showing the antibacterial activity of PNIPAM-based hydrogels with different Ag:G weight ratios (0.5:1, 1:1 and 5:1) are denoted as Ag0.5G1, Ag1G1 and Ag5G1, respectively, against *E. coli* (**a**) and *S. aureus* (**b**). Reprinted with permission from ref. [41]. Copyright 2021 Advanced Functional Materials.

The surface modification of graphene derivatives with metal compounds such as Ag, ZnO, or TiO_2_ has been adopted as a strategy to enhance the inherent antimicrobial potential of these nanostructures. For instance, hydrogels have been prepared by crosslinking Ag/G composites with acrylic acid and *N*,*N*′-methylene bisacrylamide at different mass ratios [41], leading to PNIPAM-based hydrogels. The shaking flask method [42] and the disc diffusion method [43] were applied to detect the antibacterial performance of the hydrogels against *E. coli* and *S. aureus*. The bacteria were cultured at 37 °C for 12 h, and then diluted to 10^5^ –10^6^ colony forming units per milliliter (CFU/mL). Samples with Ag:G mass ratios of 0.5:1, 1:1 and 5:1 were cut into discs and put into flasks with the bacteria suspension. The hydrogel with the optimal Ag:G mass ratio of 5:1 exhibits outstanding biocompatibility, high swelling ratio, and good extensibility, combined with very good antimicrobial activity (Figure 1). According to both antimicrobial tests, the higher the Ag content in the hydrogel, the better the antibacterial activity. Furthermore, Ag/G nanocomposites displayed much better antibacterial performance than pure Ag nanoparticles or G alone, since G can prevent the nanoparticles from aggregation. More importantly, in vivo experiments showed that this hydrogel can considerably speed up the healing rate of artificial wounds, with a wound healing ratio of 98%, higher than that of polymer mixtures such as poly(ethylene glycol)/chitosan, hence it has a potential application in wound dressing.

PNIPAM-based hydrogels containing GO and GO/CNT nanocomposites have also been prepared via dispersion of GO (or GO-CNT) in a solution of monomers followed by free radical polymerization [44]. The agar diffusion test demonstrates the good antimicrobial activity against *P. aeruginosa*.

### 3.2. Nanocomposites Based on Biocompatible Synthetic Polymers

Poly(N-vinylcarbazole) (PVK)-based materials are currently used for biomedical applications due to their antimicrobial properties. In this regard, Mejías Carpio et al. [45] developed a PVK-GO nanocomposite with only 3 wt% of GO via bulk polymerization that showed exceptional antibacterial behavior without cytotoxicity to mammalian cells. The antibacterial effects were evaluated against two Gram-negative bacteria: *E. coli* and *C. metallidurans* and two Gram-positive bacteria: *B. subtilis* and *R. opacus*. The results showed that the PVK-GO nanocomposite had stronger antimicrobial effects than raw GO. The mechanisms proposed assumed that the effectiveness of the PVK-GO in solution was due to the encapsulation of the bacteria cells by the nanocomposite, which led to reduced metabolic activity of the microorganisms and resulted, finally, in cell death.

Poly (vinyl alcohol) (PVA), a water-soluble synthetic polymer is a promising biocompatible, biodegradable, and low cytotoxic material for various medical, industrial, and commercial applications. Antibacterial PVA/G nanocomposite thin films were developed as efficient materials for food and drink packaging. Nanocomposites with 1, 5 and 10 wt% GO were prepared via a solution casting method, and their antimicrobial properties against *E. coli* and *S. aureus* were tested [46]. The films exhibited very strong antimicrobial activity against both types of bacteria, and the highest antimicrobial activity was found at the highest GO loading tested. Furthermore, they seemed to be more effective against the Gram-positive bacteria. A possible explanation for this would be the difference in their membrane structure. As is already known, Gram-negative bacteria cells possess an additional peptidoglycan layer located between the outer and the cytoplasmic membrane, thus comprising a thicker cell wall. Therefore, it is reasonable to assume that this extra layer provides an additional barrier, for *E. coli* cells, against the penetration of the biocide [47]. Recently, a one-step chemical reduction route has been employed for the synthesis of PVA nanocomposites with Ag nanoparticles anchored on GO by a solution casting technique. The nanocomposites exhibited very effective antibacterial activity against *E. coli* and *S. aureus* due to synergistic effects [48].

**Figure 2:**
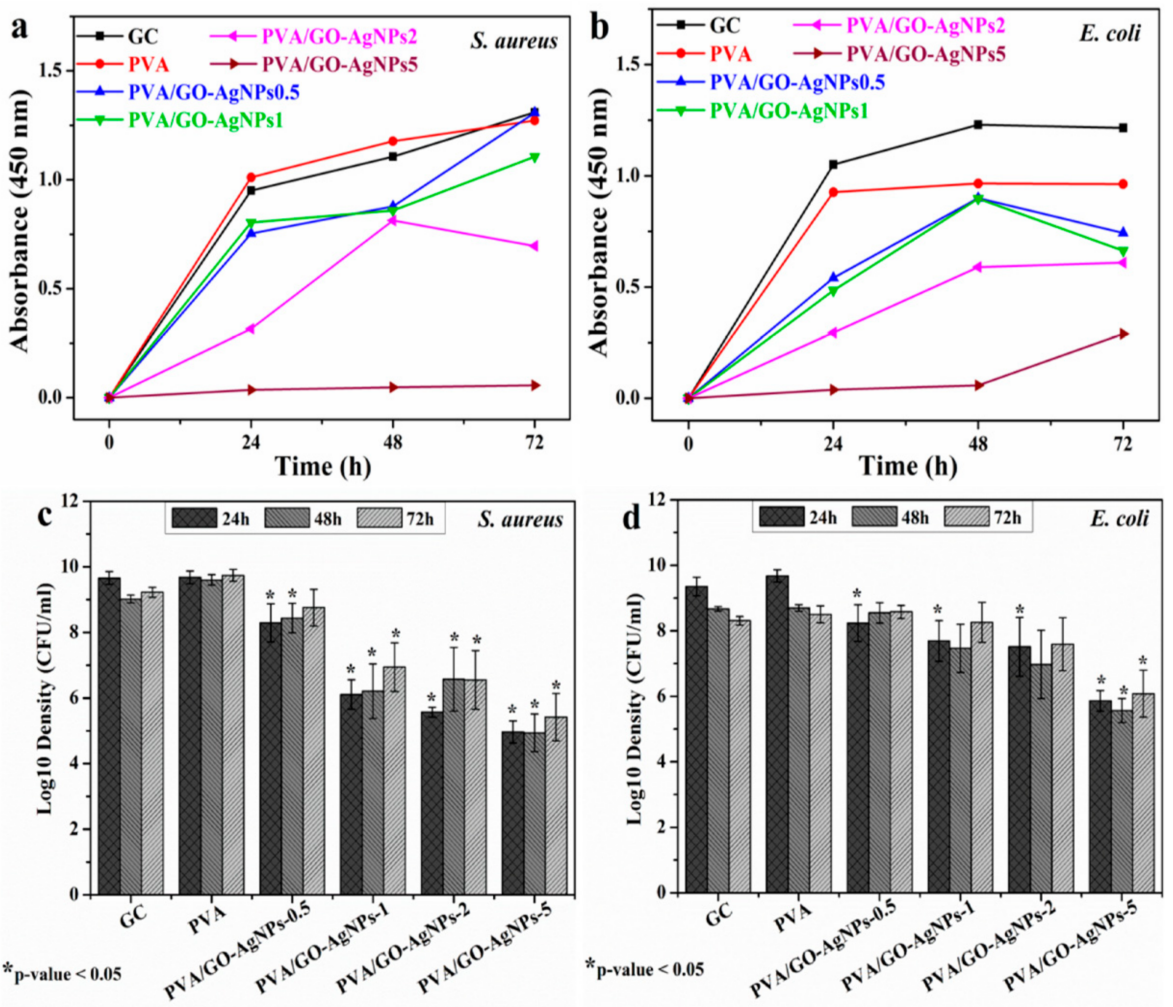
Absorbance vs. time curves for *S. aureus* (**a**) and *E. coli* (**b**) after exposure to PVA/GO-AgNPs composite films. Viable counts of *S. aureus* (**c**) and *E. coli* (**d**). Samples are labelled as PVA/GO-AgNPsx, where x denotes the wt% of the Go-AgNPs filler. Reprinted with permission from ref. [49]. Copyright 2021 MDPI.

Furthermore, the antibacterial properties of PVA/GO/Ag nanocomposite films prepared via solution blending followed by film casting were found to be time- and GO-Ag loading-dependent [49]. Composites with GO-AgNPs loadings of 0.5, 1, 2 and 5 wt% were prepared. For the antibacterial tests, *S. aureus* and *E.coli* bacteria were cultured on a plate count agar for 24 h at 37 °C, following the disk diffusion method [43]. The antibacterial activity was tested in 96-well microliter plates with an initial bacterial concentration of 5 × 10^6^ CFU/mL, using a microplate reader at 450 nm. Samples with GO-Ag concentration ≤ 1 wt% showed no inhibition of *S. aureus* cells over 72 h (Figure 2a). By increasing their loading up to 2 wt%, a drop in bacterial growth was found after 24 h of exposure, and with 5 wt% GO-Ag, a complete inhibition was attained after 24 h of exposure. In contrast, the incorporation of 0.5 and 1 wt% GO-Ag results in a small reduction in *E. coli* bacterial growth upon 24 h of exposure, and beyond that time, no inhibitory effect is observed (Figure 2b). The growth of *E. coli* is completely suppressed after 24 and 48 h of exposure of PVA films filled with 5 wt% GO-AgNPs. After 72 h this sample shows reduced antibacterial activity. Overall, it is found that the *E. coli* strain is systematically more resistant than *S. aureus* to these PVA/GO-AgNPs hybrid nanocomposites, in agreement with the results reported by other authors [45].

Another strategy consisted of the addition of starch and GO combined with Ag nanoparticles [50]. Starch acted as a reducing agent, reducing GO to rGO, from an exfoliated state to intercalated state in PVA, hence leading to an improved distribution into the PVA matrix. Thus, the antibacterial effect was a result of the synergistic effects of GO and Ag into PVA in the presence of starch.

PLA is one of the most commonly used synthetic biodegradable polymer for medical applications, though it has some shortcomings such as poor barrier properties. It has been combined with different G-based nanomaterials and their mixtures with nanoparticles, resulting in nanocomposites with higher antibacterial activity against *S. aureus* and *E. coli* [51,52,53,54]. Various factors, such as the nanocomposite preparation method, nanofiller concentration, morphology as well as degree of dispersion of the nanofiller into PLA matrix, had an influence on the final properties. Nanocomposites of PLA with either GO or thermally reduced GO have been prepared by melt mixing [51]. Both types of composites inhibited the attachment and proliferation of both bacteria, the efficiency increasing with increasing loading, and it was significantly improved by applying an electrical stimulus. GO loaded with ZnO nanoparticles was also prepared and mixed with PLA by the solution-blending method [52]. UV-visible spectra and antibacterial testing results showed that the nanocomposite films had strong UV-resistance and antimicrobial activity at very low GO-ZnO loading. Shen et al. [54] used GO/Ag hybrids as nanofillers in a PLA matrix. Nanocomposites were prepared via two processes: in situ polymerization and direct mechanical blending, and the effects of the nanofiller content and preparation method on the antibacterial properties were explored. The antibacterial efficiency increased from 0 to 99% as the loading increased up to 2.0 wt%, and composites prepared by the in situ polymerization method showed better efficiency. It was reported that GO improved the antibacterial activity of Ag nanoparticles into PLA due to better Ag-cell wall interactions, due to the good distribution of Ag onto GO surfaces, and the good dispersibility of the hybrids, which was improved by in situ polymerization method.

PVDF is a non-biodegradable, biocompatible, flexible, and inexpensive polymer widely used for water purification processes, though it suffers significantly from organic and biofouling. Many efforts have been devoted to the development of PVDF-based nanocomposite membranes with antifouling properties by adding nanomaterials. To enhance the antimicrobial resistance of PVDF-GO films, Ag nanoparticles can be incorporated. In this regard, PVDF-Ag-GO fiber mats were synthesized via electrospinning [55]. GO content was fixed at 1 wt% while the AgNPs contents were kept at 0.5, 1 and 2 wt%, relative to the weight of PVDF. The antibacterial activity of the composites was tested versus *E. coli* and *S. aureus* via the disk diffusion method, with an initial bacteria concentration of 5 × 10^6^ CFU/mL. After incubation at 37 °C for 18 h, the inhibition zones were recorded with a digital camera and measured.

**Figure 3:**
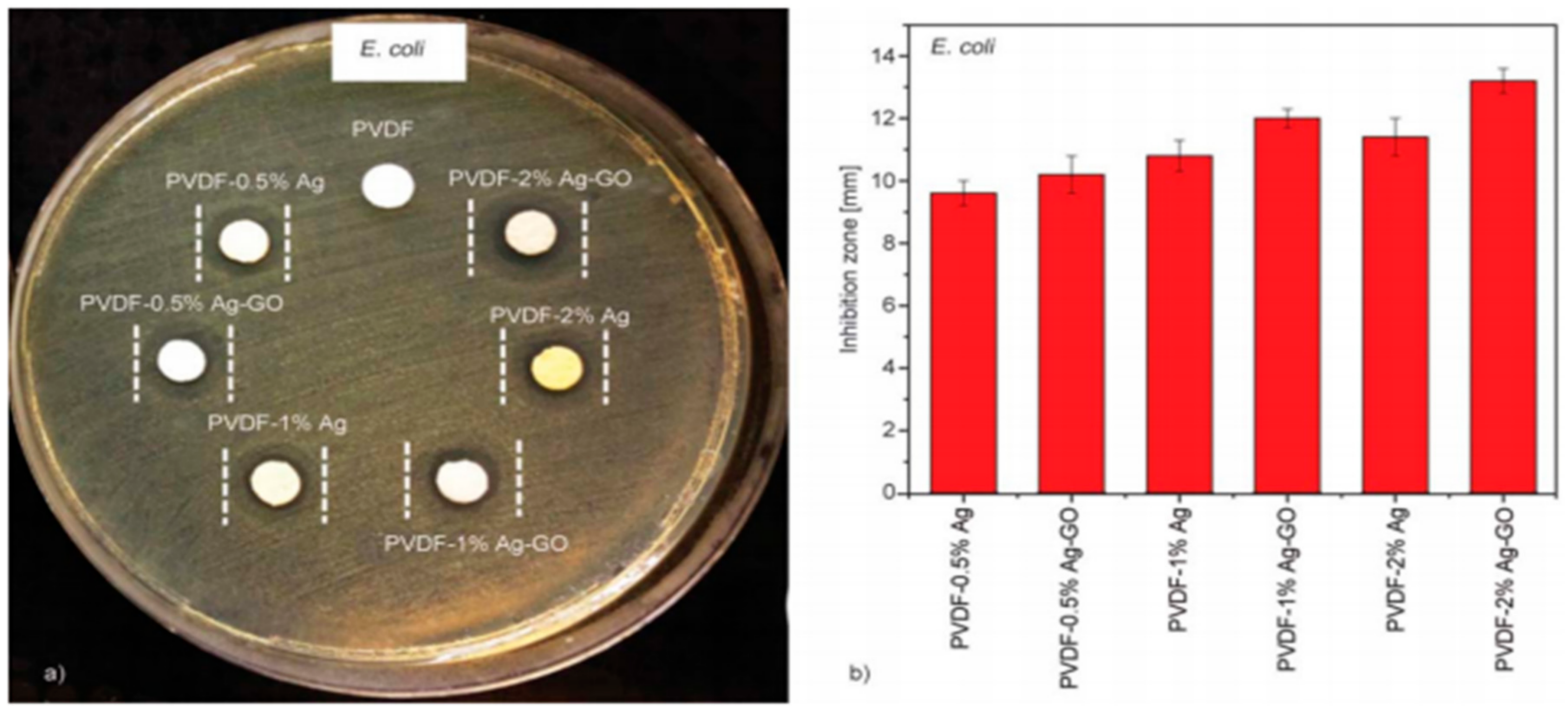
(**a**) Photograph and (**b**) diameter of the zone of inhibition of electrospun PVDF/Ag/GO nanocomposite membranes with different Ag-GO loading exposed to *E. coli*. GO content was fixed at 1 wt% while the AgNPs contents were kept at 0.5, 1 and 2 wt%. Reprinted with permission from ref. [55]. Copyright 2021 Express Polymer Letters.

Pure PVDF shows no inhibition zone, indicative of poor antibacterial activity (Figure 3). By adding 0.5% Ag to PVDF, an inhibition zone with a diameter of 9.6 mm was observed. It is known that biofilm formation arises from the premature adhesion of bacteria, and their subsequent proliferation and colonialization on the membrane surface. Consequently, hydrophobic PVDF membrane favors the bacteria adhering onto its surface. However, PVDF-Ag membranes can inhibit bacteria from attaching to the membrane surfaces by releasing Ag^+^ ions that can interact with thiol groups of enzymes of DNA, thus disrupting the metabolic processes and affecting the DNA’s ability to replicate [56]. The incorporation of 1 wt% GO into PVDF/Ag further enhances their bactericidal activity, especially against *E. coli* (Figure 3), and the best performance is attained at 2 wt% Ag loading. This behavior is ascribed to the more uniform dispersion of the Ag nanoparticles in the presence of GO, together with the sharp edge insertion mechanism, also named as the “nano-knife” effect. GO can cut the cell membrane and cause the leakage of intracellular constituents, thereby leading to bacteria death. This effect is more effective for *E. coli* with a single cell wall of peptidoglycan layer than for *S. aureus*. Other studies have demonstrated that there is a synergistic effect between the antibacterial activities of both nanofillers [57]. The presence of GO favors direct cell-nanoparticle contact, promoting the release of silver ions and hence the amount of cellular uptake.

PCL is another biodegradable synthetic polymer that does not show antibacterial activity, but, upon blending with rGO/Ag, it shows biocide action. PLC/rGO/Ag (94:5:1) hybrids prepared via solution casting yielded better performance than binary PLA/Ag or PLA/rGO [58], attributed to direct contact killing by mechanical rupture of the bacterial cells via well-dispersed rGO/Ag mixtures together with the slow and steady release of Ag ions.

Polyethylene glycol (PEG), a biocompatible and biodegradable polymer widely used for the development of tissue engineering scaffolds, has great potential for functionalizing G and its derivatives for medical applications. A few studies have assessed the biological activity and cytotoxicity of G and GO covalently modified with PEG and its derivatives [59,60]. For instance, ternary hybrids of PEG-functionalized GO with Ag nanoparticles have been prepared by a simple, fast and green microwave irradiation route [61], at different irradiation times and their bactericide properties were investigated against *S. aureus* and *E. coli* bacteria as model organisms, following the disk diffusion method [43]. The initial bacterial concentration was set at 10^5^ CFU/mL. After incubation at 37 °C for 25 h, the inhibition zones were measured.

**Figure 4:**
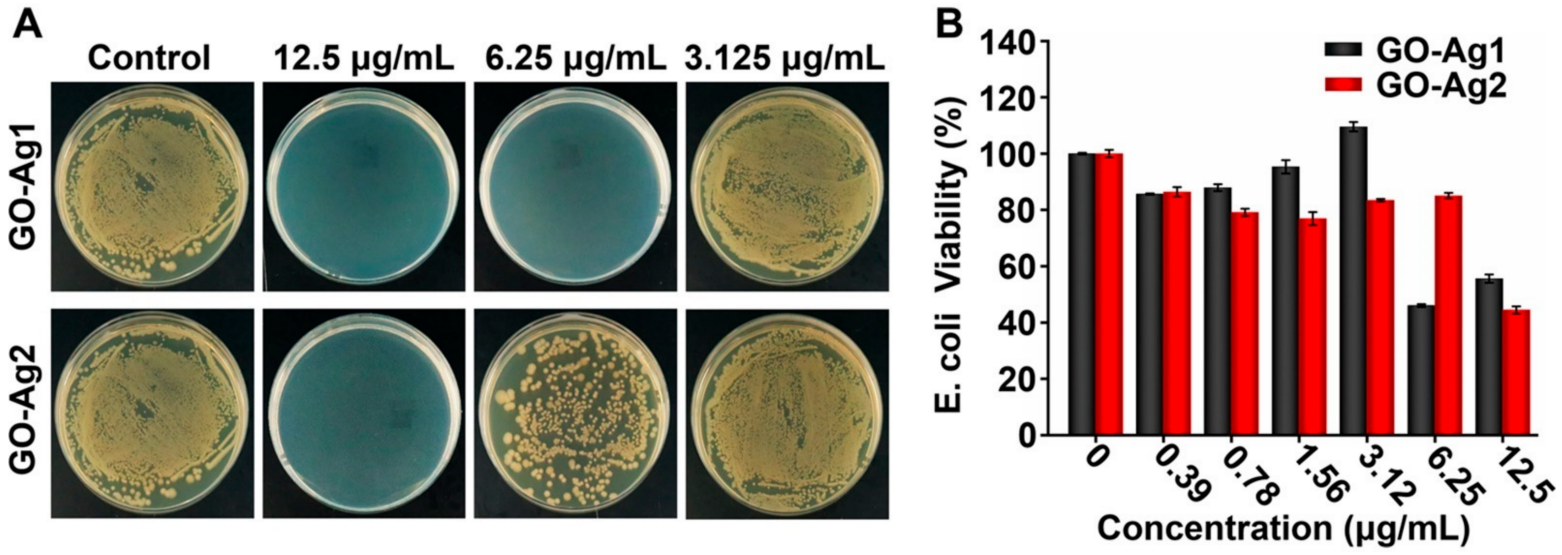
Antibacterial activity of PEG/GO/Ag nanocomposites. (**A**) Bacterial colony formation of *E. coli* under different treatments. (**B**) Bacterial cell viability of *E. coli* exposed to nanocomposites with different sizes of Ag nanoparticles: GO-Ag1 (8 nm) GO-Ag2 (50 nm). Reprinted with permission from ref. [61]. Copyright 2021 Springer Nature.

The hybrids showed excellent biocide action against *E. coli* (Figure 4), and it was found those with the smaller Ag nanoparticles (8 nm), showed better antibacterial activity than those with bigger nanoparticles (50 nm). The ternary hybrids exhibited synergistic antimicrobial activity via three effects: (a) The capping effect of GO: adsorption of the nanocomposite to the bacteria is the most important step in killing or inhibiting bacterial growth. It is believed that bacteria could be wrapped by the thin sheets of PEG/GO/Ag nanocomposites via intermolecular forces. Furthermore, the outer membrane of bacteria contains sugars, phosphates, and lipids, which can form hydrogen bonds with the functional groups of GO and subsequently form agglomerates. Bacterial nutritional supplementation and metabolism will be restricted once the bacteria are trapped by the nanocomposites. (b) The hole effect of Ag nanoparticles: small nanoparticles (8 nm) with positive surface potential can easily be adsorbed onto the bacterial surface, which can affect the permeability of the cell membranes and cause their breakage. (c) The insertion mode of action of GO: this nanomaterial can weaken the *E. coli* cell wall to some extent, and further enhance the cell membrane rupture ability of AgNPs, highlighting the significant synergistic antibacterial effect of both nanofillers.

Polyesters based on fumaric acid have recently gained a lot of attention for medical applications due to their good biocompatibility and biodegradability [62]. Among them, the most extensively explored is poly(propylene fumarate) (PPF), a linear copolyester with two ester bonds and one unsaturated carbon-carbon double bond as repeating units. However, neat PPF does not have antimicrobial activity. In this regard, PPF-based nanocomposites reinforced with GO noncovalently functionalized with PEG have been prepared via sonication and thermal curing, [36], and their antibacterial activity was tested. For this purpose, the nanocomposites with GO loading in the range of 0.1–3 wt% were submerged in a 3-day old nutrient broth of 2.0 × 10^6^ CFU/mL. After incubation at 37 °C for 48 h, the number of viable microorganism colonies was counted, and the results were expressed as mean CFU/sample. The antibacterial activity was calculated as: log(viable cell count_control_/viable cell count_composite_), where a beaker with bacteria and without nanocomposite was taken as control.

**Figure 5:**
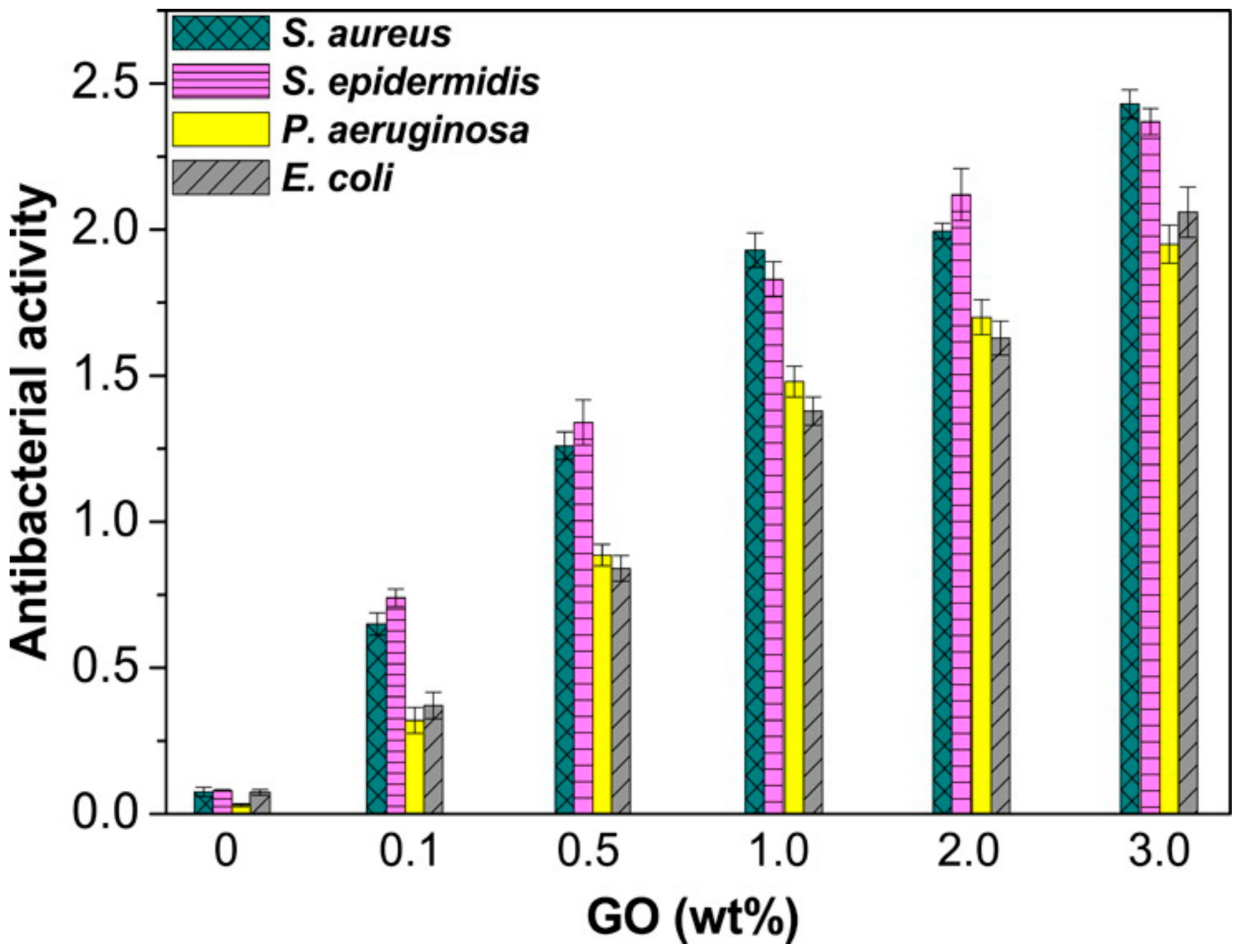
Antibacterial activity of PPF and PPF/PEG-GO composites with GO loadings of 0.1, 0.5, 1, 2, and 3 wt% against *S. aureus, S. epidermidis, P. aeruginosa* and *E. coli*. Antibacterial activity was calculated as: log(viable cell count_control_/viable cell count_composite_). Reprinted with permission from ref. [36]. Copyright 2016 American Chemical Society.

It was found that the antibacterial action against *S. aureus, S. epidermidis, P. aeruginosa* and *E. coli*. rises sharply upon increasing GO concentration (Figure 5), and the best antibacterial activity is found for the nanocomposite with 3.0 wt% GO loading. Systematically, the biocide effect is stronger versus Gram-positive cells. Accordingly, for this type of bacteria, effective antibacterial activity is already reached at 1.0 wt% GO content, while for the Gram-negative, the inhibition is only effective for the nanocomposites with 3 wt% GO loading. Moreover, no significant difference is found between the activity toward *S. aureus* and *S. epidermidis*, and the same applies for *E. coli* and *P. aeruginosa*, demonstrating that the antibacterial behavior is mainly related to the different characteristics of the cell wall between the two types of bacteria. It was proposed that GO nanosheets can produce ^•^OH radicals that attack the CO groups of the peptide linkages of the bacterial cell wall and harm the cellular components such as lipids, proteins and DNA, causing the death of the bacteria. Furthermore, composites with GO content ≤ 2.0 wt% were found to be nontoxic against normal human dermal fibroblasts, with cell viability values in the range of 90–96% after 24 h of incubation.

Novel biodegradable poly(glycolic acid-*co*-propylene fumarate) (PGA-*co*-PPF) copolymers have also been synthesized via ring-opening polymerization, and GO and hydroxyapatite nanorods have been incorporated into PGA-*co*-PPF through electrospinning to yield hybrid nanocomposite fibers [63]. The neat copolymer did not display any antibacterial activity towards bacteria, while nanocomposites with GO exhibited activity versus both *E. coli* and *S. aureus*, the bactericidal effect being systematically higher against *S. aureus*. Furthermore, the effect strongly increased with increasing nanofiller loading. It was proposed that GO nanosheets produced hydroxyl radicals that attacked the CO groups of the peptide linkages of the bacterial cell wall and harmed the cellular components. On the other hand, binary nanocomposites with HA did not show antibacterial activity versus *S. aureus,* and only a slight biocidal effect against *E. coli*. Thus, the results indicate that the mixture of HA and GO boosts the antimicrobial effect.

### 3.3. Nanocomposites Based on Natural Polymers

Several articles dealing with G-based composites with polysaccharides have been reported, since they are easily synthesized and have great potential in antimicrobial applications. In this regard, starch, chitosan, alginates, and cellulose have been used, each type of nanocomposite has different and unique properties. Chitosan (CS) is an aminated polysaccharide abundant in nature, produced from chitin by alkali deacetylation. It presents antibacterial and antifungal properties and has been widely studied as a natural antimicrobial agent in the pharmaceutical, cosmetic, agricultural, and food industries. Its properties are strongly influenced by molecular weight, degree of acetylation, and pH. Thus, CS deacetylation degree can be even lower than 10%, while its molecular weight can be higher than 1.0–2.5 MDa. In particular, low molecular weight and highly deacetylated CS have been found to be more antimicrobial against Gram-negative bacteria [64]. In this regard, a mixture of poly(lactide-*co*-glycolide) (PLGA) and CS electrospun fiber mats were functionalized with GO-Ag nanoparticles via a chemical reaction between the carboxyl groups of GO and the primary amine groups on the PLGA-CS fibers [65]. The nanocomposites effectively inactivated both Gram-negative *E. coli* and *P. aeruginosa* and Gram-positive *S. aureus* bacteria.

Nanocomposites of CS and rGO have been produced by a green methodology. The rGO was hydrothermally reduced in the presence of caffeic acid and then dispersed into CS. The films with 20–33 wt% of rGO showed an increase of inhibition in the range of 54% to 82% after 8 h of incubation [66] and this activity can avoid the oxidation of packaged foodstuffs. The antimicrobial properties were attributed to their capacity to induce cell membrane disruption and oxidative stress. Nanofilms of CS with GO and TiO_2_ nanoparticles have also been developed, and it was found that when their ratio was 20:1:4, exhibited 99% antibacterial activity against *A. niger* and *B. subtilis* [67]. The mechanism was investigated by fluorescence and morphological characterizations and seemed to be related to the damage of cell walls and cell membranes due to nanocomposite exposure.

**Scheme 4:**
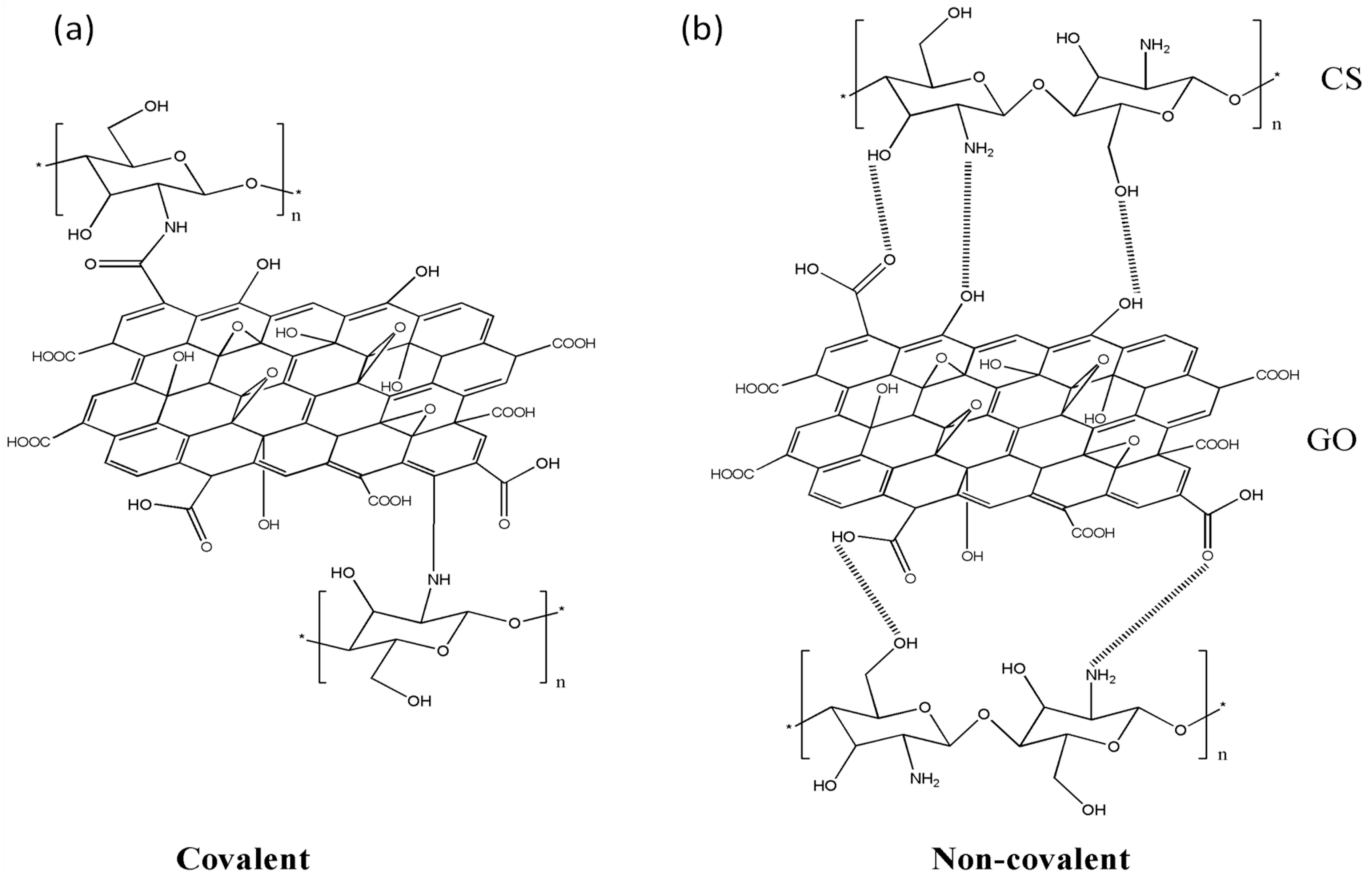
Representation of the interactions between graphene oxide (GO) and chitosan (CS): covalent (**a**) and non-covalent (**b**).

In another study, CS was combined with polyvinylpyrrolidone (PVP) and GO nanosheets to yield nanocomposites in which GO and CS can interact via covalent and non-covalent bonding [68]. Thus, the epoxy groups of GO can react with the amino groups of CS via nucleophilic substitution reaction, similar to the cross-linking and curing reaction of epoxy resins (Scheme 4a). Additionally, they can interact via H bonding between the OH and COOH groups of GO and the NH_2_ and OH groups of CS (Scheme 4b).

It was found that when the content of GO was low, it reacted with CS in the form of a single filler, with low degree of crosslinking and obvious defects. In addition, the plane and sheet structure surface of GO made the chain segment of chitosan easier to slide. In contrast, when the content was high, GO appeared in the form of aggregates that would lead to the decrease in the physical properties of the composites [69]. Improved bactericidal capacity was found against *S. aureus* and *E. coli*, in particular for the nanocomposite 25 vol% PVP and 1 wt% GO [68]. Despite the fact that the mechanism behind the antimicrobial effect of CS has not yet been determined, it is likely related to its ability to enter the bacteria cell wall through pervasion and formation of a polymer membrane on the wall surface, thus preventing nutrients from going into the cell, hence disturbing the bacteria physiological activity. Moreover, its positively charged NH_2_ groups can interact with negatively charged cell membranes, leading to the leakage of proteins and other intracellular constituents. Additionally, this biopolymer can alter the phospholipid bilayer structure in the cell membrane, changing its permeability, hence provoking the release of cellular components.

GO can improve CS antibacterial activity through a synergistic effect on bacterial inhibition by damaging the membrane cell. The mechanism of this could be related to an improvement of the surface roughness and hydrophobic character, which increases the interaction between CS and the bacteria. The nanocomposites can adsorb on the cell wall surface and bind to metals of the nutrient needed for the bacterial growth, provoking the death of the microorganism. The antibacterial action of CS-GO is somewhat more vigorous against Gram-positive bacteria.

**Figure 6:**
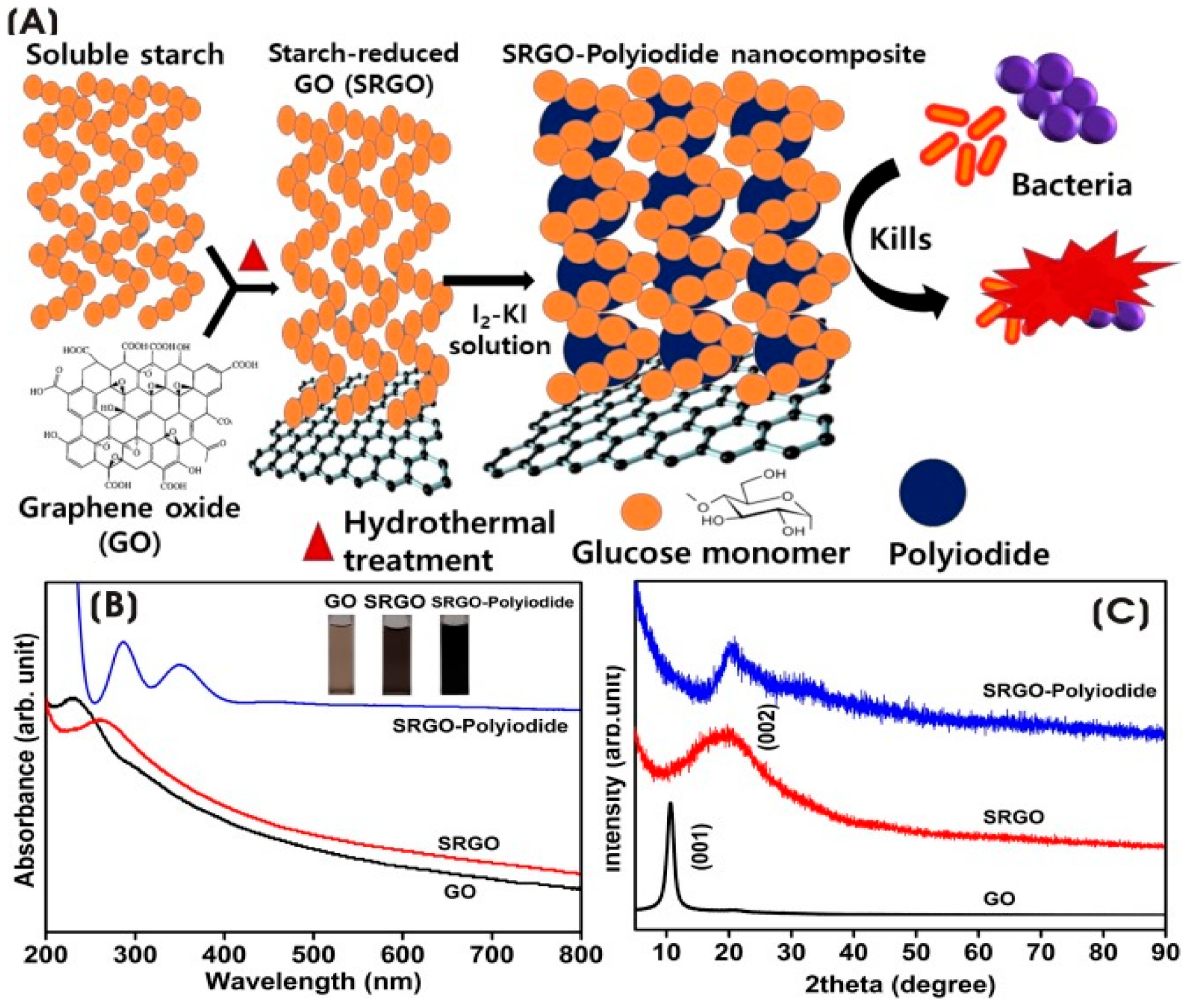
(**A**) Schematic representation of the synthesis of starch-reduced GO (SRGO)–polyiodide nanocomposite. (**B**) UV-vis spectra and (**C**) XRD pattern of GO, SRGO and the nanocomposite. Reprinted with permission from ref. [71]. Copyright 2021 Elsevier.

Starch is a mixture of two polymers: the mostly linear amylose, α-d-(1,4)-linked glucopyranosyl units, and the highly branched amylopectin, α-d-(1,4)-linked glucopyranosyl units partially substituted by α-d-(1,6) linkages units, with a proportion of 20–30% and 70–80%, respectively, depending on its source. It is non-toxic, eco-friendly, and inexpensive. However, its weak barrier properties, moisture sensitivity, and poor mechanical properties limit its use for practical purposes [70]. In a recent study [71], starch was used as a reducing agent for the preparation of starch-reduced GO (SRGO) via a hydrothermal treatment with 0.2% (wt/v) of soluble starch from potato and 0.5 mg/mL of GO. Then, the helical structures of amylose units of the starch were linked to polyiodide (a triiodide ion, I_3_^−^ prepared by reaction of I_2_ and KI) to form a SRGO–polyiodide nanocomposite (Figure 6A). UV-visible spectroscopy and X-ray diffraction analysis confirmed the presence of polyiodide in SRGO (Figure 6B,C).

Their bactericide properties were tested versus *S. aureus* and *E. coli* bacteria, following the disk diffusion method [43]. The initial bacterial concentration was set at 1.5 × 10^8^ CFU/mL. SRGO or SRGO–polyiodide (40 mg/mL) was added to the wells and incubated at 37 °C for 16 h, and then the inhibition zones were measured. The nanocomposite exhibited good antibacterial activity against *E. coli* and *S. Aureus*, with minimum bactericidal concentration values of 2.5 and 5 mg/mL, respectively, for the indicated bacteria, and inhibition zones of 22.2 and 20.2 mm, respectively, whereas neat SRGO did not exhibit any inhibition. These antibacterial starch-based nanomaterials are potential vehicles for the sustained release of polyiodide, which destroys bacteria membrane, meaning they can be suitable for sustainable food packaging applications.

Alginates (AG) are ionic-block copolymers of polysaccharides that occur in the cell wall of brown algae. They comprise regions of successive *β-*d-mannuronic acid monomers (M-blocks), regions of *α-*l-guluronic acid (G blocks), and regions of scattered M and G units [72]. The physical properties of alginates depend on the M/G ratio and the distribution of M and G units across the polymer chain. Owing to their hydrophilicity, biocompatibility, biodegradability, nontoxicity and low-cost in comparison with other biopolymers, they show great potential for the fabrication of nanocomposites with G-derivatives [73].

Marti et al. [74] crosslinked AG with Ca^2+^ cations and GO by mixing ZnCl_2_ aqueous solution with GO/AG aqueous solution under stirring followed by solvent casting. The incorporation of 1% GO into AG-based films provided strong antibacterial activity against *S. aureus* and *S. epidermidis*, and no cytotoxicity for human cells. A similar approach was applied to synthetize sodium alginates with GO, which exhibited higher antibacterial action against the two indicated bacteria for the same GO content [75].

One of the most important and industrial biopolymer polysaccharides is cellulose, a linear homopolymer of *β-*d-glucose monomers covalently linked by 1→4 glycosidic bonds that has many applications in edible coatings, paper packaging, and bio-absorption [76]. It is the foremost structural component in plant cell walls. Moreover, some bacteria and algae synthesize cellulose. However, due to its infusibility and insolubility, it is usually converted into derivatives to make it more processable. Some frequent derivatives of cellulose include ethers such as methyl cellulose and hydroxyl-ethyl cellulose, and esters such as cellulose acetate, cellulose butyrate, cellulose acetate-butyrate, etc. Nonetheless, these frequently have a slower degradation rate and their performances rapidly decline in the presence of moisture poorer performance. In order to improve cellulose properties, bionanocomposites can be synthesized. In this regard, GO-modified membranes of cellulose incorporating Ag nanoparticles were prepared in a two-step process by the in situ method. The presence of GO enhanced the deposition of Ag nanoparticles due to the electrostatic interaction between the positively charged silver ammonia complex and negatively charged oxygenated functional groups of GO. The presence of GO considerably decreased the release of Ag^+^ and the leaching of Ag nanoparticles into the aqueous solution. The produced composite membranes exhibited strong antibacterial activity against *S. aureus* and *E. coli* [77].

Agar or agar-agar is another polysaccharide with gel-forming properties widely employed in the food industry. It is a mixture of two components: the linear polysaccharide agarose, and a heterogeneous mixture of smaller molecules called agaropectin. To provide antibacterial activity, nanofillers can be incorporated in the agar matrix. Thus, an agar/rGO-PEG-Ag-ZnO nanocomposite hybrid was developed [78] via room temperature solution processing, leading to ZnO and Ag-NPs being distributed uniformly over rGO nanosheets. This nanocomposite showed high biocide activity (about 95%) against *S. aureus* and *P. aeruginosa* bacteria, attributed to a synergistic effect of rGO, Ag and ZnO nanoparticles. The higher contributor to the antimicrobial action was Ag, which prevented nutrient transportation to the cells.

Furcellaran (FUR) is a group of sulfated high molecular weight polysaccharides with gel-forming properties, obtained from red algae Furcellaria lumbricalis, that shows antibacterial activity against *S. enterica*. To expand its range of antimicrobial activity, nanocomposite films based on FUR, GO, carbon quantum dots (CQDs) and maghemite nanoparticles (MAN), have been prepared by the solution casting method [79]. The nanocomposites showed a strong inhibitory effect on the growth of *S. aureus* and *E. coli.*

Polyhydroxyalkanoates (PHAs) are natural biopolymers produced from renewable resources by using microorganisms as intracellular carbon and energy storage compounds. Their synthesis usually happens during fermentation under nutrient-limiting conditions with excess carbon. There are two main types of PHAs, short chain length PHAs (scl-PHAs) and medium chain length PHAs (mcl-PHAs). The most widely studied and easiest to yield member of this family is poly(3- hydroxybutyrate) (PHB), an isotactic, high-molecular-weight polyester with physical properties similar to those of polypropylene [80,81]. Furthermore, its copolymerization with 3-hydroxypentanoic acid yields poly(3-hydroxybutyrate-*co*-3-hydroxyvalerate), commonly known as PHBV, a biodegradable, nontoxic and biocompatible plastic used as an alternative for non-biodegradable synthetic polymers.

PHBV films with 1 wt% GO nanosheets or carbon nanofibers (CNFs) were prepared by solution casting with the aim of enhancing thermal behavior, wettability and antibacterial activity against *S. aureus*. The results showed that both nanomaterials produced similar enhancements of the physical properties. However, PHBV/GO exhibited higher antibacterial activity than that of PHBV/CNFs [82]. Gouvea et al. [83] reported the preparation of PHBV/rGO-ZnO hybrid material at 3, 6 and 9 wt% nanofiller content by melt extrusion. GO-ZnO composite was first synthesized by the simultaneous reduction of zinc diacetate and GO, at 20:1 ratio. Significant antimicrobial surface property against *E. coli* was found because of direct contact between bacteria cells and the hybrid’s surface.

**Figure 7:**
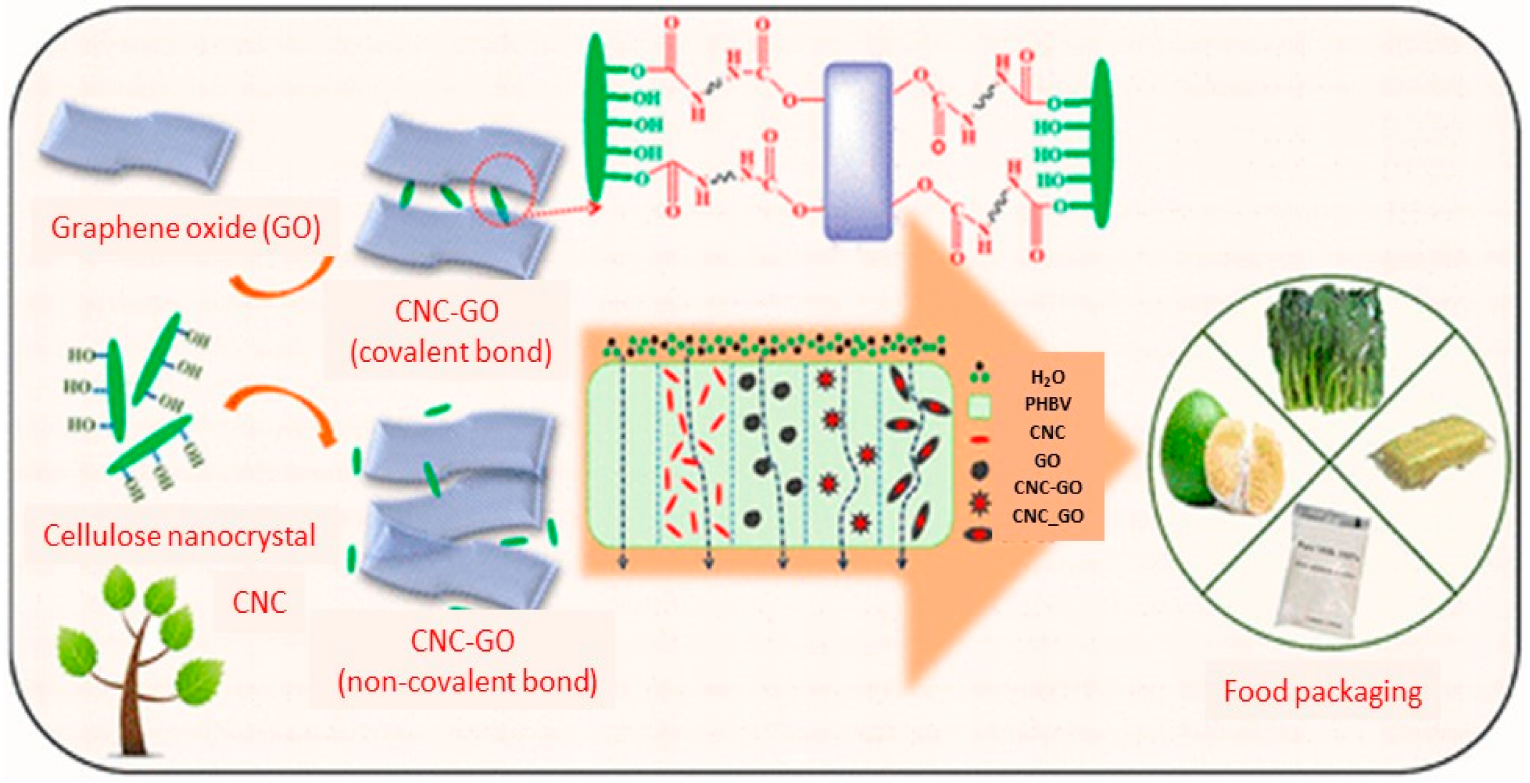
Schematic representation of the interactions between GO and cellulose nanocrystals via covalent and non-covalent bonds. Reprinted with permission from ref. [84]. Copyright 2019 American Chemical Society.

Recently, Li et al. [84] prepared novel ternary PHBV/cellulose nanocrystals (CNC)-GO nanohybrids with 1:0.5 and 1:1 wt% CNC:GO via a simple solution casting method. The synergistic effect of CNC with GO obtained by chemical grafting (CNC-GO, covalent bonds) and physical blending (CNC/GO, noncovalent bonds) on the physicochemical properties of PHBV nanocomposites was evaluated (Figure 7), and the results compared with a single nanofiller (CNC or GO) in binary nanocomposites. To evaluate their antibacterial activity using the agar diffusion method, they were submerged in a broth of 1 × 10^6^ CFU/mL. After incubation at 37 °C for 12 h, the number of viable microorganism colonies was counted. The antibacterial activity was calculated as: N_0_-N/N_0_, where N_0_ and N are the average number of colonies on neat PHBV and the nanocomposites, respectively.

No inhibitory zone was observed in neat PHBV or the binary nanocomposite with 1 wt% CNC. In contrast, a significant inhibition zone appeared around the binary nanocomposite with 0.5 wt% GO, attributed to the disruption of the cell wall of bacteria. The calculated antibacterial activity for this nanocomposite was 99.7% and 99.8% against *E. coli* and *S. aureus*, respectively. Even better properties were obtained for the ternary PHBV/CNC/GO (98:1:1 wt%) nanocomposite prepared via covalent bonding, which exhibited an antibacterial ratio of 100% against both bacteria. This result was attributed to several factors: (1) the GO damaged the bacterial cell membranes through oxidative stress or free radicals. (2) Bacteria isolated from the normal environment reduced its ability to extract nutrients needed for growth, and the combined effects resulted in cell death. (3) The ternary nanocomposite with 1 wt% CNC and GO had a compact structure and favorable interfacial adhesion between both nanofillers. Such a synergistic effect yielded high-performance ternary nanocomposites with great potential for bioactive food packaging materials.

A summary of the properties of polymeric nanocomposites with graphene-based nanomaterials is provided in Table 1.

## 4. Applications for Polymeric Nanocomposites with Graphene Materials

Currently, graphene-based polymeric nanocomposites designed to inhibit bacterial growth are being explored for numerous technological applications. For example, they are being investigated for water purification, for the fabrication of antibacterial food packaging, protective clothing, as antifouling agents, as well as several biomedical applications including bandages and wound dressing, tissue engineering, drug delivery and for the prevention of biofilm formation on medical equipment and implantable devices. Some of these applications are briefly described below.

### 4.1. Water Purification

Graphene and its derivatives have been widely explored for water treatment, in particular for the production of new filtration membranes. This is an emerging field of research which has drawn extensive attention after the work by Nair et al. [85]. However, the complex preparation process of G-based adsorbents and the difficult collection of G sheets during the adsorption process limit their practical applications. To solve these issues, G-based polymeric membranes with specific adsorption characteristics have been developed. These composite materials can be employed for different applications such as ultrafiltration, nanofiltration, forward or reverse osmosis desalination, wastewater treatment, as well as radioactive metal recovery from seawater [86,87,88]. The antibacterial and anti-fouling properties of G can also prevent corrosion and be impermeable to acids, depending on its microstructure. The precise distance between layers of carbon can be designed to allow some molecules to pass and retain others depending on their size; and for this reason, they have been applied for desalination and the elimination of pollutants.

For instance, GO/polyether sulfone (PES), reduced GO (rGO)/PES and polyethyleneimine (PEI)-coated GO membranes have been developed via a facile blending method for wastewater filtration [89]. Zeng et al. prepared PVDF membranes that were covalently functionalized GOQDs by a hydrothermal reaction. They displayed excellent antibacterial activity against *E. coli* and antibiofouling performances [90].

Maio et al. [91] used a one-step wet electrospinning method for the fabrication of polycaprolactone (PCL)/GO membranes for wastewater treatment. Chen et al. [92] recently developed an ultrafiltration membrane based on GO and polysulfone coated with polydiacetylene that exhibited strong antibacterial activity against *E. coli*. A composite elastic membrane based on G-rubber silicone has also been developed for water treatment [93], and it efficiently inhibited bacterial attachment. More complex membranes comprising GO, Ag, and a metal-organic framework (MOF) incorporated in PES have also been prepared with excellent antibacterial properties due to synergistic effects [94].

### 4.2. Antibacterial Food Packaging

Graphene materials with antibacterial properties can be employed for food packaging. Its exceptional tensile strength makes G a very attractive candidate for the development of active packaging material for food safety and preservation because its addition to films considerably increases the durability of the material and simultaneously reduces weight. Multiple examples of smart packaging films made with biopolymers and graphene-based materials have been reported in the literature [95]. The addition of G typically improves the mechanical, thermal, barrier and antimicrobial properties of the resulting nanocomposites. For instance, CS/GO nanocomposites prepared by a green method have good mechanical and barrier properties [66]. Furthermore, cross-linked GO with CS inhibited the growth of both *E. coli* and the Gram-positive B. subtilis, and simultaneously exhibited a mechanical strength and thermal stability suitable for food packaging [96]. Ghanem et al. [97] modified GO with hydrophobic poly(4-vinylbenzyl chloride) to facilitate its dispersion into a polystyrene matrix. The nanocomposite showed higher thermal stability, improved mechanical properties, and lower water vapor permeability than unmodified polystyrene, as well as biocide effect on pathogenic bacteria.

Antimicrobial polymeric films have also been synthesized via the incorporation of GO nanosheets and clove essential oil into PLA via solution casting [98]. The addition of GO into PLA matrix improved the flexibility of the composite films by lowering glass transition temperature, as well as the oxygen permeability and porosity. In addition, the developed composite film showed excellent antibacterial activity against *S. aureus* and *E. coli*. All the mentioned features indicate a high potential of graphene and GO for use as active packaging material for food safety and preservation. Konwar et al. [99] combined iron oxide-coated GO with CS hydrogel to develop nanocomposite hydrogels for applications in the food industry. The nanocomposites were fabricated via co-precipitation followed by gel casting technique. Significantly improved thermostability, tensile strength and Young’s modulus, as well as antimicrobial activity against *S. aureus*, *E. coli*, and *C. albicans* was found.

### 4.3. Protective Textiles

Graphene polymeric nanocomposites have also been used in the textile industry [100]. Their addition to fabrics has been reported to improve antibacterial activity as well as other key properties such as mechanical strength, conductivity, flame resistance, UV protection and gas barrier. Owing to these characteristics, they are good candidates for the fabrication of personal protective equipment. A few works have shown that G-based fabrics inhibit bacterial metabolism. For instance, cotton and cotton/nylon fabrics mixed with small amounts of GO or rGO showed noteworthy antibacterial activity against Gram-positive (100% and 98.4% versus *S. aureus* and *E. faecalis*, respectively) and Gram-negative bacteria (84.8% and 96.4% inhibition against *E. coli* and *P. aeruginosa*, respectively) [101]. Synthetic fabrics such as polyester, polyester doped with rGO/Ag nanocomposites and GO/PVA prevented bacterial growth [102].

### 4.4. Wound Dressings

G-based materials may also have applications in wound healing, including keeping the wound environment moist, accelerating wound closure, diminishing infections, and stimulating appropriate healing without scratch formation. In this regard, hydrogels have been prepared by crosslinking Ag/G mixtures at different weight ratios with acrylic acid and *N*,*N*′-methylene bisacrylamide. The hydrogel with the optimal Ag:G ratio of 5:1 exhibited a high swelling ratio, excellent antimicrobial ability, and good biocompatibility with outstanding mechanical properties, leading to faster wound healing process [41]. On the other hand, PU/siloxane/GO network was fabricated by the sol–gel method, and displayed excellent antimicrobial effects against Gram-negative/-positive bacterial and fungal species [103]. At the concentration of (5.0 wt%) GO, the wound dressing showed good biocompatibility with fibroblast cells, and improved the wound healing relative to collagen deposition, vascularization, and re-epithelization.

### 4.5. Tissue Engineering

Polymeric nanocomposites with G have been successfully employed in many tissue engineering investigations due to their mechanical stability. For instance, a 3D scaffold has been prepared by combining GO with CS to analyze the potential of bone tissue engineering [104]. These scaffolds showed excellent biocompatibility. The presence of GO improved the mechanical characteristics, pore formation, and bioactivity of the scaffold, and thus promoted the possibilities for in vitro and in vivo bone tissue engineering. A similar scaffold was prepared by mixing gelatin with GO and CS via a freeze-drying approach, and showed better physiochemical properties when tested for properties such as biodegradation, wettability, protein adsorption, and biomineralization, which all favored application toward bone tissue engineering [105].

### 4.6. Drug Delivery

Strong effort has been focused on using G for drug delivery. The extremely high surface area (2600 m^2^/g), and flexible structure of G enable bioconjugation and molecular loading [106]. Thus, GO is very effective for cancer therapy, including insoluble drugs [58]. For instance, Ma et al. [107] fabricated a hybrid of GO/polysebacic anhydride (GO/PSA) nanocomposite and explored its drug release efficacy for levofloxacin, a bactericidal drug. The GO/PSA hybrids exhibited a considerable increase in the release time of the drug compared to the neat polymer, as well as outstanding biocidal efficacy without any cytotoxic effect. Additionally, a PCL-based fiber containing an antibacterial agent (chlorhexidine) (CHX) and G nanoplatelets was prepared by melt spinning. The ternary fibers displayed outstanding antimicrobial activity and played a key role in minimizing the risk of infections after surgery [108]. Similarly, a ternary biocomposite with PLA, ciprofloxacin (a biocide) and G nanoplatelets was prepared, and the results showed that the incorporation of GNP increased the stiffness of the matrix and decreased the burst release outcome [109].

**Figure 8:**
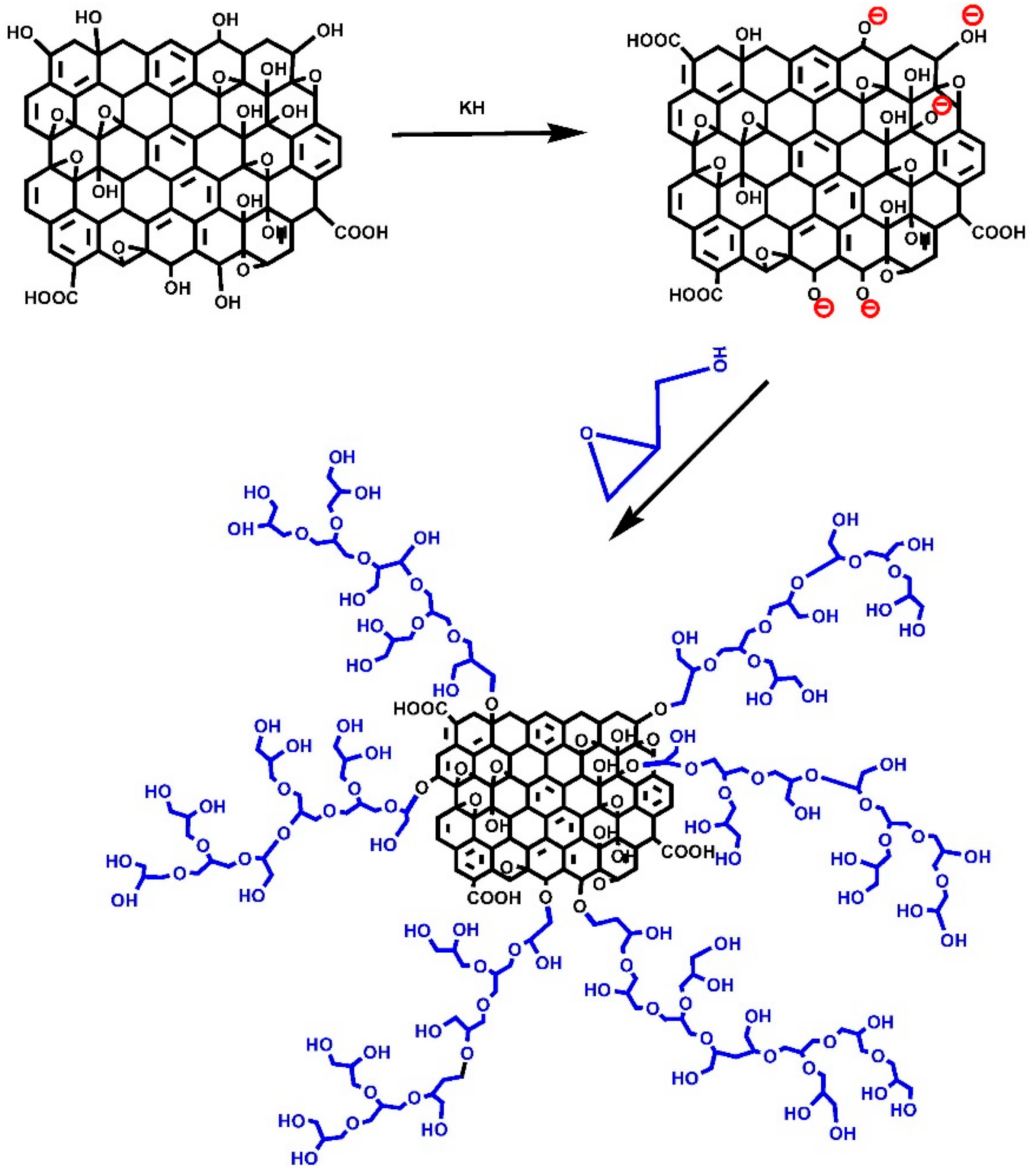
Schematic representation of the synthesis of HPG-GO hydrogel. Reprinted with premission from ref. [112]. Copyright 2021 Elsevier.

On the other hand, G-based hydrogels also have great potential for drug delivery applications. For instance, polyaspartic acid (PAp) crosslinked by G and poly(acrylamide-*co*-acrylic acid) P(AM-*co*-AA) hydrogels have been developed, and Ag, CuO and ZnO nanoparticles were incorporated within the network [110]. The hydrogels were applied to a load and controlled release of curcumin, and they showed antibacterial activity against *E. coli* and *S. aureus*. Furthermore, the physicochemical properties of GO, such as good aqueous dispersibility and colloidal stability, render it a versatile material for drug delivery. Recently, a hybrid of polyethyleneimine (PEI), PEG and folic acid covalently functionalized on GO was synthesized as a nanocarrier system for targeting hepatocellular carcinoma [111]. Hyperbranched polyglycerol-GO (HPG-GO) hydrogel was also synthesized using anionic ring-opening polymerization (Figure 8) and employed as an anticancer drug carrier [112]. This hydrogel was biocompatible and capable of delivering drugs such as amphetamines.

## 5. Conclusions

Graphene-based nanomaterials show great potential as antimicrobial agents. Up until now, many questions remain unanswered, such as the mechanisms of action, the effect of nanomaterial size, concentration, level of functionalization and so forth in the inhibition of bacterial proliferation and adhesion. This review summarizes the state of art in the preparation of graphene-based nanocomposites with antimicrobial function as new tools to tackle the current challenges in fighting against bacterial targets. Representative examples have been provided with the aim of shedding light on the range of possible mode of actions, trying to promote a better understanding on the antibacterial capabilities of polymeric nanocomposites with graphene-based nanostructures owing to synergistic effects. A classification of the different nanocomposites into acrylic and methacrylic matrices, biodegradable synthetic polymers and natural polymers has been carried out. In addition, the most important applications of these antimicrobial nanocomposites have been discussed.

In most cases, the antimicrobial properties have been tested against *E. coli* and *S. aureus* as model pathogens. Taking into account the growing spread of antibiotic-resistant bacteria related to their danger towards public health worldwide, it is crucial to explore other pathogenic species to illustrate the comprehensive range of bactericidal properties of graphene-based nanomaterials. Despite a few studies on *P. aeruginosa* and *B. subtilis* being published, a deeper understanding is necessary to obtain accurate knowledge about the antibacterial ability of this family of nanomaterials. Moreover, a more detailed and consistent understanding of the mechanisms of antimicrobial action is essential, as well as an understanding of how different parameters, such as the nanomaterial synthesis method, its defect content, lateral dimensions, shape, number of layers as well as amount of functional groups, influence the nanomaterial antibacterial properties.

On the other hand, approaches to synthesize G on a large scale and at a relatively low fee are in high demand. Despite noteworthy efforts having been carried out in this direction, current methods are seriously limited by their low efficiencies, which should be addressed for commercial applications. A reliable method for meeting the strong demand for G or its derivatives through an environmentally friendly approach and with high yield is still lacking. Another issue is that the real specific surface area of G-based nanomaterials is significantly lower than the predictions due to the strong agglomeration tendency of the sheets via hydrophobic interactions, and this limits their antibacterial activity. In this regard, novel strategies to effectively exfoliate G in a green way are pursued.

Another limitation for the practical application of this type of nanocomposites is that currently, no in vivo antibacterial activity study has been performed on animal models. While graphene-based materials may be valuable for wound healing and other biomedical applications, future uses in vivo cannot be ruled out. One important question that has to be answered first is whether G-based nanomaterials can selectively target pathogenic microorganisms without affecting normal mammalian cells or non-pathogenic bacteria. Very scarce studies related to the selective killing of pathogenic microorganisms have been published. Furthermore, the toxicity of G-based nanomaterials is not clear yet. Despite considerable efforts in assessing the effect of these nanomaterials on human health and the environment, results are often inconsistent. They might provoke cytotoxicity in humans, and this matter should be elucidated. It is important to note that the effects of G-based materials may differ depending on their intrinsic properties.

While the connection to the biomedical market is still missing, researchers are continuously proposing different graphene-based materials as antibacterial agents. Thus, some works on the antibacterial properties of graphene quantum dots (GQDs) have been highlighted. GQDs display lower toxicity than GO and cause no apparent toxicity in vivo. Further GQDs suspensions can produce ROS upon photo-excitation, hence can be used for the photodynamic treatment of pathogens, a strategy scarcely developed up to date.

Overall, the development and use of antimicrobial polymeric nanocomposites with G is hindered by the lack of techniques to provide simple, reproducible, and cost-effective nanomaterials at a large scale. The research in this field is still in its infancy. The combination between these carbon nanostructures and the polymers has opened new properties and applications due to their synergistic effects. Nonetheless, more work is needed to ensure that the developed nanocomposites are easy to manufacture and have no toxicity. It can be envisaged that after wide-ranging research in the field and continuous innovative efforts, nanocomposites incorporating polymers and G-based nanomaterials could provide a new outlook for the development of antimicrobial agents. This milestone can be reached via collaboration between different disciplines and technologies (i.e., nanotechnology and polymer science).

## Figures and Tables

**Table 1:** Characteristics of polymer nanocomposites with G-based nanomaterials.

Matrix	Nanofiller	Processing Method	Bacteria	Inhibition	Comments	Reference
	(wt%)			(%)		
PMMA	GO-Ag (1) GO-Ag (2)	SN + curing	*E. coli*	-	Synergistic effect of both nanofillers	[38]
			*S. aureus*			
			*S. mutans*			
PMMA fibers	GO (8)	Pressurized gyration	*E. coli*	85	GO well distributed across fiber	[40]
PNIPAM	Ag/G (0.5:1)	In situ polymerization	*E. coli* *S. aureus*	-	Synergistic effect: G prevented Ag aggregation	[41]
	Ag/G (1:1)					
	Ag/G (5:1)					
PNIPAM	GO/CNT (1:1)	FRP	*P. aeruginosa*	-	Strong interaction GO-PNIPAM	[44]
PVK	GO (3)	Bulk polymerization	*B. subtilis*	89	Better properties than raw GO	[45]
			*R. opacus*	89		
			*E. coli*	89		
			*C. metallidurans*	91		
PVA	G (1)	SN+ solution casting	*E. coli*	92	5 wt% G is the optimal nanofiller ratio	[46]
	G (5)		*E.coli*	95.8		
	G (10)		*E.coli*	97.1		
	G (1)		*S. aureus*	92.3		
	G(5)		*S. aureus*	99.6		
	G(10)		*S. aureus*	99.7		
PLA	GO/Ag (2)	In situ polymerization	*E. coli*	99	Improved Ag dispersion with GO	[54]
			*S. aureus*	99		
PVDF fibers	GO/Ag (1:0.5)	ES	*E. coli* *S. aureus*	-	GO promotes release of Ag^+^	[55]
	GO/Ag (1:1)					
	GO/Ag (1:2)					
PCL	GO/Ag (5:1)	SN+ solution casting	*E. coli*	59	Rupture of the cells by rGO-Ag	[58]
PPF	PEG-GO (3)	SN+ curing	*S. aureus*	97	Strong interaction between PEG-GO and PPF	[36]
			*S. epidermidis*	94		
			*P. aeruginosa*	85		
			*E. coli*	81		
CS	GO/TiO_2_ (4/16)	SA	*A. niger*	99	Synergistic effect of all components	[67]
			*B. subtilis*	99		
AG	GO (1)	SN+ solution casting	*S. aureus*	99	Strong AG-GO interactions	[74]
			*S. epidermis*	99		
AGAR	rGO/Ag/ZnO	solution casting	*S. aureus*	95	Uniform distribution of Ag and ZnO over rGO	[78]
			*P. aeruginosa*	95		
PHBV	CNC/GO (1:1)	solution casting	*E. coli*	99.7	Synergistic effect by chemical grafting	[84]
			*S. aureus*	99.8		

SN: sonication; FRP: free radical polymerization; ES: electrospinning; SA: self assembly.
